# Development of One-Tube Multiplex Arbitrary (RAPD and ISSR) Marker-Based SCAR Assay for Simultaneous Detection and Authentication of Indian Senna (*Senna alexandrina* Mill.) and Its Adulterant Species

**DOI:** 10.3390/ijms27073165

**Published:** 2026-03-31

**Authors:** Sarika Chouksey, Pushkar Kaira, Maneesha Pandey, Asghar Ali, Mohd Ashraf Ashfaq

**Affiliations:** 1 Department of Botany, School of Chemical & Life Sciences, Jamia Hamdard, New Delhi 110062, India; 2 Biochemistry Discipline, School of Sciences, Indira Gandhi National Open University, New Delhi 110068, India; 3 Department of Biochemistry, School of Chemical & Life Sciences, Jamia Hamdard, New Delhi 110062, India

**Keywords:** authentication, multiplex PCR, SCAR, *Senna alexandrina* Mill., *Cassia fistula*, *Senna sophera*

## Abstract

Indian senna (*Senna alexandrina* Mill.), a perennial medicinal species belonging to the family Fabaceae, holds significant therapeutic and commercial importance owing to its rich content of sennosides and rhein derivatives, which confer well-established laxative properties. Its high market demand, however, renders the species vulnerable to deliberate or inadvertent adulteration. While previous investigations have utilized functional marker systems such as SCoT (Start Codon Targeted Polymorphism)- and CBDP (CAAT Box Derived Polymorphism)-derived SCAR (Sequence Characterised Amplified Region) markers for genetic characterization, the present study is the first to report the development of sequence-specific RAPD- and ISSR-based SCAR markers consolidated into a single-tube multiplex PCR assay. Genomic DNA isolated from young leaves of *S. alexandrina* and its commonly encountered adulterant species was amplified using RAPD primer OPI-02 and ISSR primer UBC-835. Polymorphic amplicons were cloned, sequenced, and employed for the design of SCAR primers, which were rigorously validated for specificity. Species-specific SCAR markers were successfully integrated into a single multiplex reaction, enabling precise and unequivocal identification of *S. alexandrina*, *Cassia fistula* and *Senna sophera*. The multiplex amplification profiles were entirely consistent with corresponding uniplex assays, endorsing the method’s robustness and reproducibility. This streamlined, one-tube multiplex SCAR-PCR system represents a significant advancement toward reliable, high-throughput molecular authentication of Indian senna and its closely related medicinal plant species (adulterants).

## 1. Introduction

*Senna alexandrina* Mill., commonly known as Indian senna, is a well-established medicinal plant of the family Fabaceae, widely valued for its potent laxative properties and long-standing use in the management of constipation. This fast-growing shrub is extensively cultivated across several regions of India, where its leaves, pods, and seeds are integral to Unani medicine and are also recognized in various international pharmacopeias. Phytochemical investigations have identified more than 28 bioactive compounds in different plant parts [1,2], predominantly anthraquinones and their glycosides. Among these, sennosides A and B are regarded as the principal therapeutically active constituents and are of considerable pharmaceutical significance [3]. Further, Indian senna leaves are traditionally employed to address conditions such as anorexia, hepatomegaly, and other gastrointestinal and systemic disorders [4]. While deeply rooted in traditional Indian medicine, the plant also enjoys substantial global recognition. In the United States, it is classified as a Category III stimulant laxative and approved by the FDA for over-the-counter use. India remains the foremost global producer, contributing more than 70% of total exports. In addition to its purgative action, Indian senna demonstrates anti-inflammatory, antioxidant, antimicrobial, antiviral, and potential anticancer properties, and is used in the management of skin ailments, jaundice, and even as a natural hair colorant [5,6,7].

The genus *Cassia* encompasses approximately 350 species, each contributing uniquely to ecological sustainability and traditional medicine. A substantial proportion—nearly 70%—of *Cassia* species are native to the Americas, while about 13% are distributed in Australia and 10% in Africa; the remaining species occur across Southeast Asia, the Pacific Islands and parts of north-eastern regions [8]. This broad geographic distribution underscores the genus’s ecological adaptability and medicinal relevance. *Cassia fistula* Linn., commonly referred to as Indian laburnum or Amaltas, is a prominent member of the family Caesalpiniaceae. It occupies a significant position in Ayurvedic, Unani, and traditional Chinese systems of medicine, where it is employed for both preventive and therapeutic applications. The leaf extract of *C. fistula* demonstrates notable antitussive and wound-healing properties, and the plant exhibits a wide spectrum of pharmacological activities supported by contemporary investigations [9,10]. Likewise, *Senna sophera* Linn., widely known as “Kasondi,” holds recognized therapeutic value in Unani medicine. It is commonly distributed throughout India and other tropical regions, frequently growing in wastelands, along roadsides, and at forest margins. Classical Unani texts describe its utility in managing conditions such as epilepsy, hepatic disorders, jaundice, dermatological ailments, piles, joint pain, fever, and palpitations [11], reflecting its longstanding medicinal importance.

Genetic diversity, both within and among species, is an inherent biological phenomenon that arises through adaptation to diverse environmental conditions. The assessment of such diversity is fundamental to accurate species identification and authentication. Over the past three decades, molecular markers have become indispensable tools in this domain. Techniques such as Random Amplified Polymorphic DNA (RAPD) and Inter-Simple Sequence Repeat (ISSR) markers have been widely adopted for the characterization and authentication of species. RAPD markers were initially employed to identify disease-resistance genes in lettuce [12,13,14,15,16,17], while ISSR markers were introduced by Zietkiewicz et al. (1994) [18] for the analysis of cultivated plant species [19,20,21,22]. Despite their utility, arbitrary marker systems such as RAPD and ISSR are often limited by issues of poor reproducibility. This limitation can be effectively addressed by converting RAPD and/or ISSR-derived polymorphic fragments into SCAR markers [16,23,24,25], which are sequence-specific and yield more consistent and reliable results. In addition, PCR multiplexing has emerged as a powerful and efficient approach that enables the simultaneous amplification of multiple target sequences using different primer pairs within a single reaction. This strategy substantially reduces time, labor, and cost, while enhancing analytical throughput. Consequently, multiplex PCR has become highly valuable for applications including polymorphism detection, gene deletion analysis, mutation screening and identification of viruses, bacteria, fungi and plant species [26,27,28].

## 2. Results

### 2.1. Screening of RAPD and ISSR Primers

**Figure 1:**
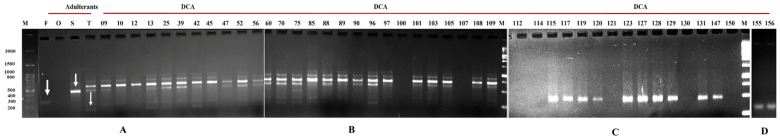
PCR profile of 48 samples using RAPD-OPI-02 primer. Lane M:100 bp ladder, Gel [**A**]: *Cassia fistula* (F), *Senna occidentalis* (O), *Senna sophera* (S), *Senna tora* (T), and accessions of Indian senna DCA9 to DCA56; Gel [**B**]: DCA60 to DCA109; Gel [**C**]: DCA112 to DCA150; Gel [**D**]: DCA155 to DCA156. Arrows represent the polymorphic amplicons. Polymorphic amplicons were obtained in *C. fistula*, and adulterants, *S. sophera* and *S. tora*.

**Figure 2:**
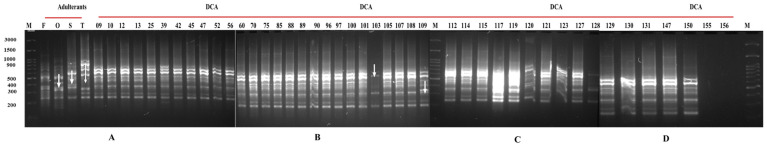
PCR profile of 48 samples using ISSR UBC 835 primer. Lane M: 100 bp ladder; Gel [**A**]: *Cassia fistula* (F), *Senna occidentalis* (O), *Senna sophera* (S), *Senna tora* (T), and accessions of Indian senna DCA9 to DCA56; Gel [**B**]: DCA60 to DCA109; Gel [**C**]: DCA112 to 128; Gel [**D**]: DCA129-DCA156. Arrows represent polymorphic amplicons. Polymorphic amplicons were obtained in *S. occidentalis*, *S. sophera*, and *S. tora*, and DCA103 and DCA109 accessions of Indian senna.

A total of 63 RAPD (Table 1) and 17 ISSR primers (Table 2) were screened using genomic DNA of all 44 accessions of Indian senna and 4 adulterant species for polymorphism. The range of amplified amplicons per primer was 100 to 3000 bp in size. Among the RAPD primers used, OPI-02 documented polymorphism in adulterant species (*C. fistula* and *S. sophera*) (Figure 1), and ISSR UBC 835 primer captured polymorphism in Indian senna accession DCA109 (Figure 2). RAPD OPI-02 and ISSR UBC 835 primers both clearly distinguished the adulterants *Cassia fistula* and *Senna sophera* species, and Indian senna accession DCA 109, and thus were taken forward for the development of SCAR markers.

### 2.2. Development of RAPD- and ISSR-Based SCAR Marker

#### 2.2.1. Ligation, Cloning, and Sequencing of Transformed Clones

**Figure 3:**
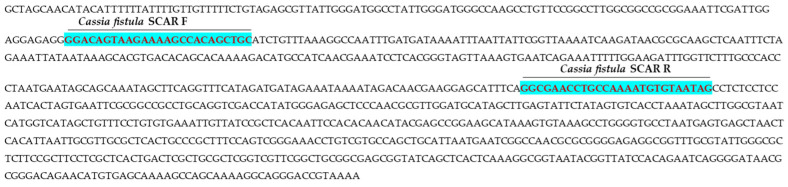
Nucleotide sequence (312 bases) (GenBank accession No. OR677404) of RAPD-based SCAR specific for *Cassia fistula*. Highlighted sequences indicate newly developed RAPD OPI-02-based SCAR forward and reverse primers (*Cassia fistula* SCAR F/*Cassia fistula* SCAR R).

**Figure 4:**
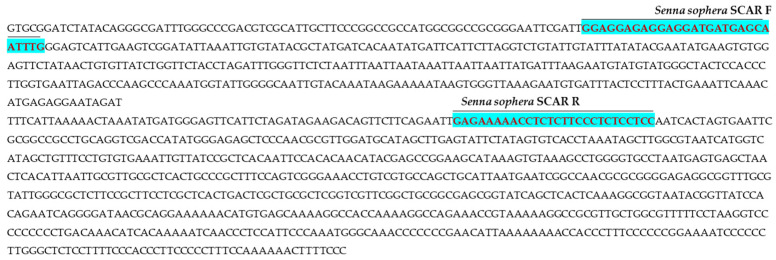
Nucleotide sequence (441 bases) (GenBank accession No. PQ642940) of RAPD-based SCAR specific for *Senna sophera*. Highlighted sequences indicate newly developed RAPD OPI-02-based SCAR forward and reverse primers (*Senna sophera* SCAR F/*Senna sophera* SCAR R).

**Figure 5:**
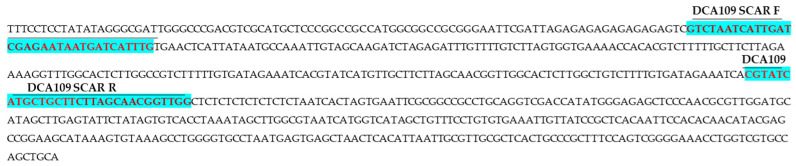
Nucleotide sequence (252 bases) (GenBank accession No. OR677403) of ISSR UBC 835-based SCAR for Indian senna accession DCA 109. Highlighted sequences indicate newly developed ISSR UBC 835-based SCAR forward and reverse primers (CA109 SCAR FP/CA109 SCAR RP).

Polymorphic DNA fragments generated by the RAPD primer OPI-02, measuring 312 bp in *Cassia fistula* and 441 bp in *Senna sophera*, as well as a 252 bp fragment from the ISSR primer UBC 835 in the Indian senna accession DCA109, were excised from the gel and purified. These amplicons were subsequently ligated into the pGEM-T Easy cloning vector and transformed into *Escherichia coli* DH5α competent cells. Recombinant clones were screened using blue-white selection. Three confirmed positive clones, two from RAPD OPI-02 corresponding to *C. fistula* and *S. sophera*, and one from ISSR UBC 835 corresponding to the Indian senna accession DCA109, were selected for sequencing. The obtained sequences were subjected to BLAST 2.14 analysis to assess homology with the existing nucleotide databases. Species-specific signature sequences of 312 bp and 441 bp were identified for *C. fistula* (Figure 3) and *S. sophera* (Figure 4), respectively, while a 252 bp sequence specific to accession DCA109 and other Indian senna accessions was obtained using ISSR UBC 835 (Figure 5). Formatting of mathematical components:

#### 2.2.2. Analysis of SCAR Marker Sequences

The BLAST analysis of the selected sequences indicated no significant similarity with existing sequences in the NCBI database, confirming their novelty. Based on these unique sequences, species-specific, monolocus, and highly reproducible SCAR (Sequence Characterized Amplified Region) primers were designed and ordered for *Cassia fistula*, *Senna sophera*, and the DCA109 accession. These primers were developed to enhance specificity and reliability for species identification. The novel sequences were submitted to the GenBank database, and accession numbers were assigned accordingly. In the case of RAPD-derived SCAR markers, the following primers were developed: *C. fistula* forward primer 5′-GGACAGTAAGAAAAGCCACAGCTGC-3′ and reverse primer 5′-CTATTACACATTTTGGCAGG-3′ (GenBank accession no. OR677404, Figure 3); *S. sophera* forward primer 5′-GGAGGAGAGGAGGATGATGAGCAATTTG-3′ and reverse primer 5′-GGAGGAGAGGGAAGAGAGGTTTTTCTC-3′ (GenBank accession no. PQ642940, Figure 4). The primer pair comprising a forward primer 5′-GTCTAATCATTGATCGAG-3′ and a reverse primer 5′-AACCGTTGCTAAGCAGCATGATACG-3′ (GenBank accession no. OR677403, Figure 5) for the ISSR-derived SCAR marker is specific to the DCA109 accession of Indian senna. Details of these primers are summarized in Table 3.

#### 2.2.3. Amplification of SCAR Markers for Validation

**Figure 6:**
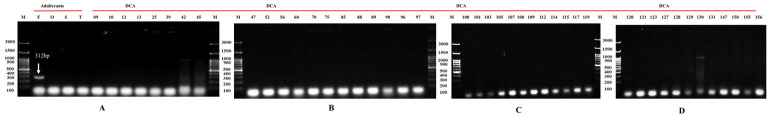
*Cassia fistula*-specific SCAR marker validation: Lane M:100 bp ladder. Gel [**A**]: Four adulterant species (*Cassia fistula* (F), *Senna occidentalis* (O), *Senna sophera* (S), and *Senna tora* (T)) and accessions of Indian senna DCA9 to DCA45; Gel [**B**]: DCA47 to DCA97 accessions; Gel [**C**]: DCA100 to DCA119; Gel [**D**]: DCA120 to DCA156. Arrows represent newly developed SCAR marker specific to *Cassia fistula*.

**Figure 7:**
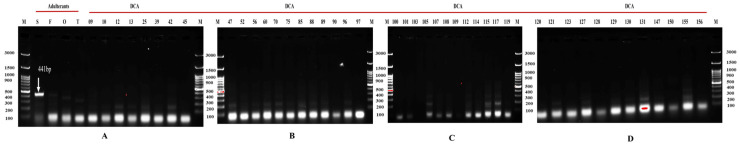
*Senna sophera*-specific SCAR validation: Lane M: 100 bp ladder. Gel [**A**]: *Senna sophera* (S), *Cassia fistula* (F), *Senna occidentalis* (O), and *Senna tora* (T) and accessions of Indian senna DCA9 to DCA45; Gel [**B**]: DCA47 to DCA97; Gel [**C**]: DCA100 to DCA119; Gel [**D**]: DCA120 to DCA156. Arrows represent the newly developed SCAR marker to *Senna sophera*.

**Figure 8:**
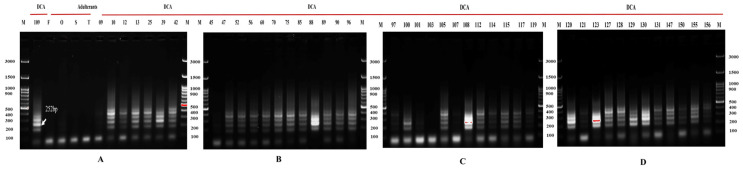
Indian senna CA109-specific SCAR validation: Lane M:100 bp ladder. Gel [**A**]: *Cassia fistula* (F), *Senna occidentalis* (O), *Senna sophera* (S), and *Senna tora* (T) and accessions of Indian senna DCA9 to DCA45; Gel [**B**]: DCA47 to DCA97; Gel [**C**]: DCA100 to DCA119; Gel [**D**]: DCA120 to DCA156. Arrows represent the newly developed CA 109-specific SCAR marker.

The newly synthesized SCAR primers were evaluated across a panel of 48 genomic DNA samples, comprising 44 accessions of Indian senna and four related adulterant species. The SCAR markers consistently yielded distinct, sharp, and reproducible amplification bands, specifically corresponding to the RAPD-derived SCAR for *Cassia fistula*, the RAPD-derived SCAR for *Senna sophera*, and the ISSR-derived SCAR for DCA109 accession, along with other accessions of Indian senna. The SCAR PCR conditions were optimized, with the annealing temperature set at 59 °C for the RAPD-OPI-02-based SCAR marker, yielding a 312 bp amplicon specific to *C. fistula* (Figure 6), 55.5 °C for the RAPD-OPI-02-based SCAR marker, producing a 441 bp band specific to *S. sophera* (Figure 7), and 52 °C for the ISSR UBC-835-based SCAR marker, amplifying a 252 bp fragment native to Indian senna accession DCA109 (Figure 8).

#### 2.2.4. Multiplex PCR with Newly Developed SCAR Markers

**Figure 9:**
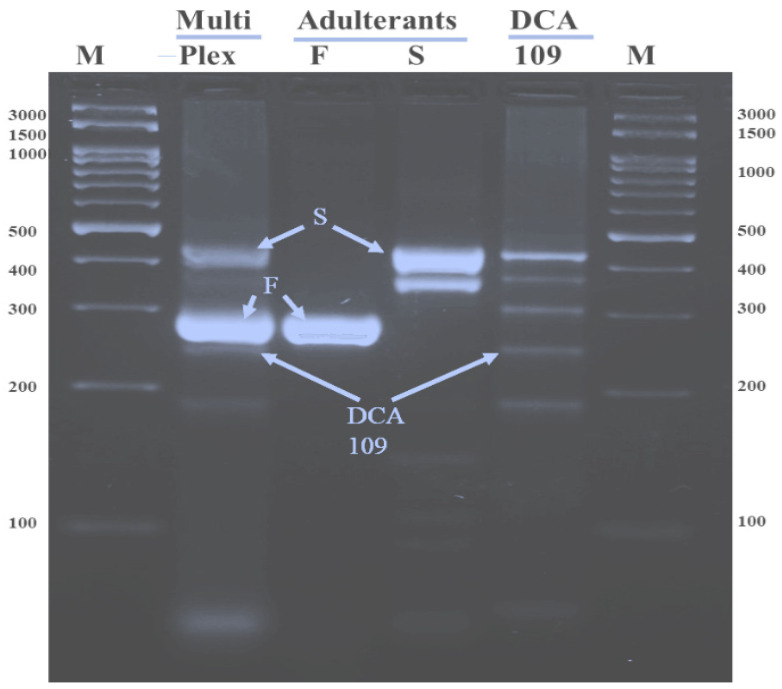
Validation of one-tube multiplex PCR: Lane M: 100 bp ladder; Lane 2: One-tube multiplex PCR gel profile of *C. fistula* (F), *S. sophera* (S), and Indian senna DCA 109; Lane 3, 4, 5: uniplex PCR gel profile of *C. fistula* (F), *S. sophera* (S), and Indian senna DCA 109 respectively.

Multiplex and uniplex PCR assays were performed using the newly developed SCAR markers to confirm their specificity and effectiveness. Each of the three SCAR primer sets, targeting *Cassia fistula*, *Senna sophera*, and the DCA109 accession of Indian senna, was specifically assayed both under multiplex and uniplex PCR conditions (Figure 9). The results obtained in multiplex PCR were species-specific signature amplicons of 441 bp identified for *S. sophera*, 312 bp for *C. fistula*, and 252 bp specific to accession DCA109 of Indian senna. The result was the same in uniplex PCR with some non-specific bands.

## 3. Discussion

The consumption of crude herbal drugs necessitates unequivocal and reliable identification of medicinal plant materials to safeguard their safety, quality, credibility, and therapeutic efficacy. Adulteration, whether deliberate or inadvertent, remains a global concern, often driven by morphological similarities among dried plant parts, inadequate regulatory oversight, and escalatings commercial demand. Such practices not only erode consumer confidence but also pose significant public health risks when adulterants or inappropriate substitutes enter the food chain. The availability of authentic herbal drugs in the marketplace is increasingly undermined by the widespread and indiscriminate sale of adulterated, substituted, or misidentified plant materials, particularly in dried or powdered forms. These challenges are further compounded when conventional identification methods prove inadequate for accurately determining the botanical identity of herbal products.

Traditional methods of plant identification are grounded in morphological, anatomical, and biochemical parameters, each offering distinct advantages as well as inherent limitations. These non-DNA-based approaches remain indispensable for preliminary screening, assessment of chemical composition, detection of processing-induced alterations, and evaluation of structural and topographical integrity. However, DNA-based authentication provides a level of precision, specificity, reproducibility, reliability, and stability that conventional methods cannot consistently achieve. By enabling unambiguous species-level identification, irrespective of developmental stage or processing condition, molecular techniques have transformed the field of plant authentication and are now widely regarded as the gold standard.

In this context, the development and use of SCAR markers, derived from RAPD and ISSR profiles, offers a highly specific, reproducible, and reliable molecular approach for authentication. Unlike RAPD and ISSR markers, which often exhibit low reproducibility, SCAR markers are based on unique DNA sequences that enable precise identification of target species. Further, one-tube multiplex PCR (assembling amplicons from multiple SCARs) offers a practical, feasible solution for authenticating multiple medicinal plant species simultaneously in a single reaction. By using species-specific primers, this technique enables rapid, reliable, and cost-effective identification of multiple plant DNAs in processed herbal products or complex mixtures, even when morphological or chemical analysis is not feasible. This combined approach of SCAR and multiplex PCR brings together the exploratory power of RAPD and ISSR with the specificity of SCAR markers and integrates them into a single, streamlined PCR reaction. This not only improves the quality control of the herbal products but also supports the conservation of endangered medicinal plant species by preventing illegal substitution and overharvesting.

A wide range of molecular techniques is routinely employed to evaluate genetic diversity through polymorphism analysis [29] and to elucidate phylogenetic relationships among species. Marker systems such as RAPD, ISSR, SCoT, and CBDP have proven particularly effective in differentiating authentic plant materials from their adulterants. Polymorphic bands generated through these approaches serve as reliable diagnostic markers and form the basis of species authentication. In this context, the development of SCAR markers by Paran and Michelmore (1993) represented a significant advancement in molecular identification of markers linked to downy mildew resistance genes in lettuce [30]. SCAR markers, being sequence-specific, offer enhanced reproducibility and specificity. They can be developed from polymorphic fragments generated through various marker systems, including RAPD [30,31,32], ISSR [33], CBDP [34], and SCoT [35]. More recently, SCAR marker analysis derived from refined SCoT and CBDP methodologies has facilitated the precise characterization and authentication of Indian senna accessions and their adulterant species collected from geographically diverse regions across India [36].

RAPD, a simple and cost-effective method, has been employed to study intra- and intergeneric genetic diversity, particularly in bamboo species [16,37]. However, Saiki et al., 1988 [38], noted that fragment polymorphisms produced by RAPD-PCR amplifications are not always reproducible. The genetic diversity of Moringa species, for example, has been successfully analyzed using RAPD and ISSR markers [19]. SCAR markers, as proposed by Paran and Michelmore (1993) [30], offer greater reproducibility and have been widely used for genotype authentication and identification. They have been developed for numerous crops, including rice [39], lettuce [30], common bean [40], wheat [41], raspberry [42], grape [43], and Brassica [44]. For example, Yuskianti et al., 2011 [45], screened 288 RAPD primers and developed 46 SCAR primers useful for genotype and clone identification in *Paraseriathes falcataria*. Similarly, Kalita et al., 2014, developed SCAR markers specific to Assam-type tea using RAPD primers [25], while Kambiranda, 2009, created SCAR markers for the authentication of *Pueraria tuberosa* [46]. Furthermore, Chaves-Bedoya and Nunez, 2007, developed a 900 bp RAPD SCAR from the OP-Y7 RAPD primer, capable of differentiating male papaya plants from female and hermaphrodite plants in three Colombian cultivars [47]. Other studies, such as those by Oyama et al., 2009, developed SCAR markers for sex determination in *T. dioica* species [48], while Patil et al., 2013, identified markers for sex determination in *Momordica dioica* using RAPD-based SCAR primers [7].

ISSR markers have also proven effective for analyzing genetic diversity in a wide range of crop species, including finger millet [49], vigna [50], rice [20], sweet potato [51], wheat [52], and plantago [53], and have also been used in genetic diversity studies of tea [54], summer squash [55], ragi [56], *C. latifolia* [57], orchid [58], and Moringa species [19]. Recently, ISSR-based SCAR markers have been utilized for plant authentication in species such as ginger [59], *Panax ginseng* [33], *Crocus sativus* [60], *Punica granatum* [31], *Ocimum tenuiflorum* [61], *Cyperus rotundus* [62], maple [23], *Trapa natans* [63], *Lycium chinense* [64], cardamom [24], *Dendrobium officinale* [65], as well as in plant-derived food and medicinal products such as *Aconitum heterophyllum*.

In multiplex PCR, the selected primer pairs must amplify distinct target DNA fragments, which is facilitated by similar optimal annealing temperatures, primer lengths greater than 30 bp, and GC content between 35% and 60%. These primers should not be internally complementary to one another [27]. However, multiplex PCR optimization presents challenges due to sensitivity and specificity.

Moon et al. (2016) successfully developed two sets of multiplex SCAR markers that enabled rapid and precise identification of *Patrinia scabiosifolia*, *P. villosa*, *P. rupestris*, and *P. saniculifolia* [66]. In a subsequent study, Moon et al. (2017) examined the complete internal transcribed spacer (ITS) sequences of six medicinal plants—*A. stricta*, *A. triphylla*, *A. triphylla* var. *japonica*, *C. lanceolata*, *C. pilosula*, and *G. littoralis* [67]. Based on the ITS1 region, they designed SCAR markers that distinguish four of these herbal species. They further established a multiplex SCAR assay, which facilitated the simultaneous identification of the selected medicinal plants in a single analysis.

Further, Kim et al. (2016) developed a rapid method for identifying *Aralia continentalis* Kitag. and *Angelica biserrata* C.Q. Yuan & R.H. Shan using ITS2 sequences combined with multiplex SCAR markers [68]. In an earlier study, Moon et al. (2015) employed RAPD) analysis to develop SCAR markers for *Akebia quinata*, *A. trifoliata*, *Aristolochia manshuriensis*, and *Clematis armandii* [69]. These markers were subsequently refined into a multiplex PCR assay, enabling simultaneous identification of all four species.

Noh et al., 2021, focused on developing SCAR markers coupled with a multiplex SCAR assay for the accurate and efficient identification of authentic *Taxilli Herba* (TH) and *Visci Herba* (VH) samples [70]. In another investigation, Noh et al. (2018) analyzed the ITS regions of nuclear ribosomal RNA genes in *Angelica dahurica*, *A. dahurica* var. *formosana*, and two closely related species, *A. anomala* and *A. japonica* [71]. Sequence variations among these species were used to design primers specific to each species. With these primers, they established and optimized a multiplex SCAR assay that enabled rapid and accurate species identification and the detection of potential adulteration in a single PCR run, eliminating the need for sequencing.

In the present study, three primer pairs were developed for RAPD- and ISSR-based one-tube multiplex SCAR assays to distinguish Indian senna, *Cassia fistula*, and *Senna sophera*. Results from both uniplex and multiplex PCR consistently yielded polymorphic amplicons at 59 °C. Among them, *Cassia fistula* displayed the strongest amplification, while *Senna sophera* displayed slightly weaker amplification along with some nonspecific products. The amplification efficiency for Indian senna DCA109 was comparatively lower, likely because product concentrations were below saturation levels. These variations in band intensity may be explained by differences in annealing and extension efficiency, or by disparities in the concentrations of reaction components between uniplex PCR and multiplex PCR [72,73]. This study is the first of its kind aimed at unambiguous, fool-proof identification of Indian senna, *Cassia fistula*, and *Senna sophera* in a one-tube multiplex PCR-based assay, facilitating high-throughput, minimal sample requirement, and rapid turnaround time, thus making it ideal for large-scale screening in quality control laboratories, regulatory agencies, and the herbal industry. Moreover, its adaptability to degraded DNA (as long as the primer binding site is intact) from powdered or dried materials further expands its utility.

## 4. Materials and Methods

### 4.1. Germplasm Collection

A total of 44 accessions of Indian senna (*Senna alexandrina* Mill.) were sourced from the ICAR-Directorate of Medicinal and Aromatic Plants Research (DMAPR), Anand, Gujarat, India. Additionally, four closely related adulterant species—*Cassia fistula*, *Senna occidentalis*, *Senna sophera*, and *Senna tora* were included for the aim of developing species-specific RAPD- and ISSR-based SCAR markers.

### 4.2. Extraction of Genomic DNA

Genomic DNA was extracted from the collected plant material, comprising 44 Indian senna accessions and the four aforementioned adulterant species, by employing a modified Cetyltrimethylammonium Bromide (CTAB) (HiMedia Laboratories Private Limited, Mumbai, Maharashtra, India) protocol as described by Doyle (1991) [74]. Both DNA purity and concentration were assessed employing a Nanodrop spectrophotometer (Thermo Scientific, Loughborough, UK) and validated using electrophoresis on a 1% (*w*/*v*) agarose (HiMedia Laboratories Private Limited, Mumbai, Maharashtra, India) gel. The DNA samples were then standardized to a concentration of 50 ng/μL and further used for downstream PCR.

### 4.3. PCR Amplification Using RAPD and ISSR Primers

A total of 63 RAPD primers (Table 1) and 17 ISSR primers (Table 2), synthesized by BioServe Biotechnologies (India) Pvt. Ltd. (Bangalore, India), were initially screened for amplification. These primers were subjected to PCR using DNA from all 44 Indian senna accessions and the four-adulterant species.

#### 4.3.1. RAPD-Primed PCR

RAPD-primed PCR was carried out in a total reaction volume of 15 μL, containing 1×PCR buffer, 50 ng of template DNA, 1.5 mM MgCl_2_, 160 μM dNTPs, 1.0 μM primer, and 0.5 U of *Taq* DNA polymerase. Amplification was achieved in a 96-well Bio-Rad T100 Thermal Cycler System, Indiaunder the following conditions: initial denaturation at 95 °C for 3 min, followed by 45 cycles of denaturation at 94 °C for 1 min, annealing at 36 °C for 45 s, and extension at 72 °C for 30 s, concluding with a final extension at 72 °C for 10 min.

#### 4.3.2. ISSR-Primed PCR

The ISSR (Table 2) primed PCR reactions were conducted in a total volume of 15 µL, comprising 50 ng of genomic DNA, 10× PCR Taq Buffer A, 25 mM MgCl_2_, 25 mM dNTPs, 10 µM ISSR primer, and 0.5 U *Taq* DNA polymerase (Bangalore Genei, Bangalore, India). The thermal cycling protocol was initiated with a pre-denaturation at 94 °C for 2 min, followed by annealing at 37 °C for 2 min, and a primer-specific annealing temperature, then extension at 72 °C for 2 min. Subsequently, 45 amplification cycles consisting of denaturation at 94 °C for 1 min, annealing at 37 °C for 1 min, and extension at 72 °C for 2 min, concluding with a final extension at 72 °C for 10 min using a Bio-Rad T100 Thermal Cycler System.

The resulting PCR products were resolved through electrophoresis on a 3.5% agarose gel in 1× TAE buffer at 150 V. Bands were identified after staining with ethidium bromide (EtBr) and visualized using a gel documentation system (Alfaimager Mini Gel Documentation System, Biocompare, CA, USA). A 100 bp size ladder (Bangalore Genei, India) was used as a reference for determining the fragment sizes.

### 4.4. Cloning and Sequencing of Specific RAPD and ISSR Amplicons

Distinct amplicons generated through PCR from Indian senna accessions and their adulterant species were resolved on agarose gels. Target bands were excised and purified using the QIAquick Gel Extraction Kit, India. The purified fragments were subsequently ligated into the pGEM-T Easy Vector System (Promega, Madison, WI, USA) and transformed into *Escherichia coli* DH5α competent cells. Recombinant plasmid clones were screened and confirmed through plasmid PCR and restriction analysis. Sequencing of confirmed clones was outsourced to Barcode Biosciences, Bangalore, India.

### 4.5. BLAST Analysis and Design of SCAR Primers

All validated sequences were subjected to BLAST analysis to assess their similarity with known nucleotide sequences available in public databases. Based on the sequence data gained from RAPD and ISSR amplicons, species-specific SCAR primers were designed using the online OligoCalc primer design tool (https://oligocalc.eu/, accessed on 10 December 2024).

### 4.6. SCAR Marker Validation

PCR validation of the designed SCAR primers was carried out in a 15 µL reaction mixture containing: 1.5 µL of 10× PCR buffer with Mg Cl_2_, 10 mM dNTPs, 10 µM forward and reverse primers, 50 ng of genomic DNA, and 2U *Taq* DNA polymerase. Thermal cycling included an initial denaturation at 94 °C for 3 min, followed by 35 cycles of denaturation at 94 °C for 1 min, annealing at primer-specific temperatures for 45 s, and extension at 72 °C, with a final extension of 10 min at 72 °C for 45 s. All 44 Indian senna accessions and two adulterant species were amplified using the newly synthesized SCAR primers. PCR products were resolved on 2% agarose gels, visualized under UV light, and documented using a gel documentation system.

### 4.7. Multiplex PCR

For multiplex PCR, the reaction mix consisted of 10 µL of PCR master mix (Takara Bio (EU), Saint-Germain-en-Laye, France), 0.2 µM forward and reverse SCAR primers of *C. fistula*, 0.5 µM forward and reverse SCAR primers of DCA 109, and 0.5 µM forward and reverse SCAR primers of *S. sophera*, 50 ng of genomic DNA from *C. fistula*, *S. sophera*, and DCA 109, and 4.6 µL of nuclease-free water, in an overall volume of 20 µL. In parallel, uniplex PCR reactions were conducted for each species individually using PCR master mix and the same concentration of SCAR primers, and the same DNA was used as in the multiplex reaction mix. The PCR reactions were set as follows: initial denaturation at 94 °C for 3 min; 35 cycles of denaturation at 94 °C for 1 min, annealing at 59 °C for 1 min, and extension at 72 °C for 45 s; followed by a final extension at 72 °C for 10 min. For validation, all three SCAR primer pairs (*C. fistula*, *S. sophera*, and DCA 109) were also amplified separately. PCR products were resolved using 4% agarose gel electrophoresis and documented via a gel imaging system.

## 5. Conclusions

The present study focuses on the development of a one-tube multiplex PCR assay based on arbitrary marker (RAPD and ISSR-derived) SCAR markers for the detection and authentication of Indian senna, *Cassia fistula*, and *Senna sophera*. In essence, three species-specific SCARs were successfully developed and integrated into a single multiplex PCR system, enabling unequivocal detection and authentication of the aforementioned species. The results obtained from both multiplex and uniplex PCR are in complete consensus. The one-tube multiplex PCR strategy described in the present study represents a significant advancement towards the rapid, precise, and high-throughput authentication of closely related medicinal plant species.

## Figures and Tables

**Table 1:** List of RAPD primers used in the study.

S. No.	Name	Sequence (5′–3′)	%GC	Tm (°C)
1.	OPA-02	TGCCGAGCTG	70.0	34.0
2.	OPA-03	AGTCAGCCAC	60.0	32.0
3.	OPA-04	AATCGGGCTG	60.0	32.0
4.	OPA-08	GTGACGTAGG	60.0	32.0
5.	OPA-10	GTGACGTAGG	60.0	32.0
6.	OPA-11	CAATCGCCGT	60.0	32.0
7.	OPA-14	TCTGTGCTGG	60.0	32.0
8.	OPA-16	AGCCAGCGAA	60.0	32.0
9.	OPA-18	AGGTGACCGT	60.0	32.0
10.	OPB-01	GTTTCGCTCC	60.0	32.0
11.	OPB-18	CCACAGCAGT	60.0	32.0
12.	OPC-02	GTGAGGCGTC	70.0	34.0
13.	OPC-03	GGGGGTCTTT	60.0	32.0
14.	OPC-04	CCGCATCTAC	60.0	32.0
15.	OPC-05	GATGACCGCC	70.0	34.0
16.	OPC-08	TGGACCGGTG	70.0	34.0
17.	OPC-17	TTCCCCCCAG	70.0	34.0
18.	OPC-18	TGAGTGGGTG	60.0	32.0
19.	OPC-19	GTTGCCAGCC	70.0	34.0
20.	OPC-20	ACTTCGCCAC	60.0	32.0
21.	OPD-01	ACCGCGAAGG	70.0	34.0
22.	OPD-02	GGACCCAACC	70.0	34.0
23.	OPD-03	GTCGCCGTCA	70.0	34.0
24.	OPD-07	TTGGCACGGG	70.0	34.0
25.	OPD-08	GTGTGCCCCA	70.0	34.0
26.	OPD-18	GAGAGCCAAC	60.0	32.0
27.	OPD-20	ACCCGGTCAC	70.0	34.0
28.	OPG-03	GAGCCCTCCA	70.0	34.0
29.	OPG-05	CTGAGACGGA	60.0	32.0
30.	OPG-18	GGCTCATGTG	60.0	32.0
31.	OPH-12	ACGCGCATGT	60.0	32.0
32.	OPI-02	GGAGGAGAGG	70.0	34.0
33.	OPN-02	ACCAGGGGCA	70.0	34.0
34.	OPN-04	GACCGACCCA	70.0	34.0
35.	OPN-05	ACTGAACGCC	60.0	32.0
36.	OPN-06	GAGACGCACA	60.0	32.0
37.	OPN-08	ACCTCAGCTC	60.0	32.0
38.	OPN-10	GTGATCGCAG	60.0	32.0
39.	OPN-11	TCGCCGCAAA	60.0	32.0
40.	OPN-12	CACAGACACC	60.0	32.0
41.	OPN-15	CAGCGACTGT	60.0	32.0
42.	OPN-16	AAGCGACCTG	60.0	32.0
43.	OPN-18	GGTGAGGTCA	60.0	32.0
44.	OPV-08	GGACGGCGGT	70.0	34.0
45.	OPT-20	GACCAATGCC	60.0	32.0
46.	OPAF-05	CCCGATCAGA	60.0	32.0
47.	OPAF-09	CCCCTCAGAA	60.0	32.0
48.	OPAF-14	GGTGCGCACT	70.0	32.0
49.	OPAF-19	GGACAAGCAG	60.0	32.0
50.	OPA-01	CAGGCCCTTC	70.0	34.0
51.	OPJ-10	AAGCCCGAGG	70.0	34.0
52.	OPX-04	CCGCTACCGA	60.0	32.0
53.	OPX-07	GAGCGAGGCT	60.0	32.0
54.	OPX-12	TCGCCAGCCA	60.0	32.0
55.	OPX-08	CAGGGGTGGA	60.0	32.0
56.	OPAP-01	AACTGGCCCC	60.0	32.0
57.	OPAP-20	CCCGGATACA	70.0	34.0
58.	OPF-05	CCGAATTCCC	60.0	32.0
59.	OPAF-15	CACGAACCTC	70.0	34.0
60.	OPA-07	GAAACGGGTG	60.0	32.0
61.	OPI-01	ACCTGGACAC	60.0	34.0
62.	OPU-10	ACCTCGGCAC	60.0	32.0
63.	OPU-20	ACAGCCCCCA	70.0	34.0

Abbreviation: S. No.: Serial Number.

**Table 2:** List of ISSR primers used in the study.

S. No.	Primer	Sequence (5′–3′)	%GC	Tm (°C)
1	UBC/ISSR 02/40	ACTGACTGACTGACTG	50	43
2	UBC/ISSR 5	GTGGTGGTGGTGGTG	66.6	47
3	UBC/ISSR 8	GAGAGAGAGAGAGAGACT	50	48
4	UBC/ISSR 11	CACACACACACACACG	56.2	46
5	UBC/ISSR 13	GTGGTGGTGGTGGTG	66.6	47
6	UBC/ISSR 16	GTGGTGGTGGTGGAC	66.6	47
7	UBC/ISSR 17	AGAGAGAGAGAGAGAGG	52.9	47
8	UBC/ISSR 21	TCTCTCTCTCTCTCTCC	52.9	47
9	UBC/ISSR 22	TCTCTCTCTCTCTCTCC	52.9	47
10	UBC/ISSR 24	TCTCTCTCTCTCTCTCG	52.9	47
11	UBC/ISSR 825	GAGAGAGAGAGAGAGAGT	50	48
12	UBC 826/10	ACACACACACACACACC	52.9	47
13	UBC 835	AGAGAGAGAGAGAGAGYC	50	48
14	UBC 840	GAGAGAGAGAGAGAGAYT	46.3	38
15	UBC 872	GATAGATAGATAGATA	25	33
16	UBC 873	GACAGACAGACAGACA	50	43
17	UBC 880	GGAGAGGAGAGGAGA	55.5	47

**Table 3:** Total number of RAPD-SCAR and ISSR-SCAR developed in this study.

Primary Primer	SCAR Primer	SCAR Primer Sequence (5′-3′)	Length (Bases)	Annealing Temperature (°C)	Amplicon Length	GenBank Accession No.
RAPD OPI-02	*C. fistula* FP	5′GGACAGTAAGAAAAGCCACAGCTGC3′	25	59°	312 bp	OR677404
	*C. fistula* RP	5′CTATTACACATTTTGGCAGGTTCGCC3′	26			
RAPD OPI-02	*S. sophera* FP	5′GGAGGAGAGGAGGATGATGAGCAATTTG3′	28	55.5°	441 bp	PQ642940
	*S. sophera* RP	5′GGAGGAGAGGGAAGAGAGGTTTTTCTC3′	27			
ISSR UBC-835	CA109 FP	5′GTCTAATCATTGATCGAGAATAATGATCATTTG3′	33	52°	252 bp	OR677403
	CA109 RP	5′AACCGTTGCTAAGAAGCAGCATGATACG3′	28			

## Data Availability

The original contributions presented in this study are included in the article. Further inquiries can be directed to the corresponding authors, and the submitted accessions and adulterant datasets are available in online repositories as well. The names of repositories and accession number(s) can be found at https://www.ncbi.nlm.nih.gov/genbank (accessed on 10 December 2024).
